# Strain-induced violation of temperature uniformity in mesoscale liquids

**DOI:** 10.1038/s41598-020-69404-1

**Published:** 2020-08-07

**Authors:** Eni Kume, Patrick Baroni, Laurence Noirez

**Affiliations:** Laboratoire Léon Brillouin (CEA-CNRS), Univ. Paris-Saclay, CEA-Saclay, 91191 Gif-sur-Yvette Cédex, France

**Keywords:** Physics, Fluid dynamics

## Abstract

Thermo-elasticity couples the deformation of an elastic (solid) body to its temperature and *vice-versa*. It is a solid-like property. Highlighting such property in liquids is a paradigm shift: it requires long-range collective interactions that are not considered in current liquid descriptions. The present microthermal studies provide evidence for such solid-like correlations. It is shown that ordinary liquids emit a modulated thermal signal when applying a low frequency (Hz) mechanical shear stress. The liquid splits in several tenths microns wide hot and cold thermal bands, all varying synchronously and separately with the applied stress wave reaching a sizable amplitude of ± 0.2 °C. Thermomechanical coupling challenges fluid dynamics: it reveals that the liquid does not dissipate the energy of shear waves at low frequency, but converts it in non-uniform thermodynamic states. The dynamic thermal changes work in an adiabatic way supporting the hypothesis of the excitation of macroscopic elastic correlations whose range is limited to several tens of microns, in accordance with recent non-extensive theoretical models. The proof of thermomechanical coupling opens the way to a new generation of energy-efficient temperature converters.

## Introduction

Control and understanding of small-scale flows is rich in potential applications. Because the laws for flow and thermodynamics reach often the frontiers of their validity at nano and microscales, the physics is difficult but fascinating. Modern techniques give us the opportunity to probe precisely physical properties at the sub-millimeter scale, to uncover new and major knowledges that challenge the concept of continuum mechanics. Low frequency (Hz) shear elasticity belongs typically to these newly identified scale-dependent properties^1–8^. It tells that liquid molecules might be long-range elastically correlated at the mesoscale. Such a claim challenges the commonly accepted understanding that liquids exhibit shear elasticity at high frequency only (typically in the megahertz–gigahertz range for molecular liquids)^9–13^. The concept of high frequency elasticity is justified by the fact that thermally activated excitations ("jumps") are limited to close neighbours due to the absence of translational symmetry in liquids. These excitations define a local relaxation time as *τ* = *η/G*, where *η* is the viscosity and *G* the shear elastic modulus. For excitations of frequency smaller than the inverse of these local short timescale (typically ω < 10^9^–10^14^ Hz for small molecules) and in absence of correlations, liquids are supposed to fulfil the hydrodynamic conditions (flow regime). The present mesoscopic study reveals instead that a low frequency (ω ~ Hz) mechanical shear stress is not dissipated in hydrodynamic conditions but it induces synchronous modulated hot and cold thermal signals in adequacy with the excitation of long-range elastic correlations. Recently, a revision of the Frenkel calculation revealed that liquid might support low frequency elastic shear waves specifying the scale conditions under which it applies (k-gap model)^14–16^. The low frequency shear elasticity is weak of the order of 1–10^3^ Pa, negligible in practise and dependent on the scale^1–4,6–8^ in agreement with the prediction of a finite propagation length^14–16^. These hidden elastic liquid molecule correlations make possible the identification of new mesoscopic liquid properties. This is the goal of the present thermal study.

**Scheme 1:**
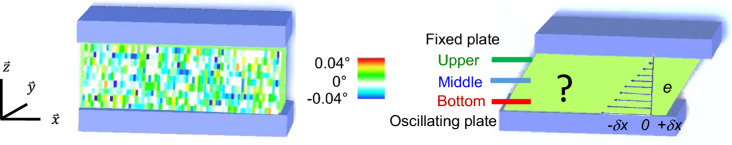
The liquid is confined between two high energy plates (Alumina surfaces). At left, the thermal image of the liquid is viewed at rest from the gap plane (xOz) (63 × 13 pixels (renormalized) thermal image of glycerol recorded at room temperature, e = 0.240 mm gap). The impact of a low frequency (~ Hz) mechanical shear strain *γ* = *δx/e*, where *δx* is the amplitude of displacement and *e* the gap thickness, on the liquid temperature is studied.

To access physical properties of a material, a general principle is to observe the response function to an external stress. Here, the liquid is submitted to a low frequency (~ Hz) mechanical shear strain (external stress) and we observe the dynamic thermal response via accessing the instant temperature on each point of the liquid gap (Scheme 1).

Different liquids have been tested. The liquids chosen here are the glycerol and the polypropylene glycol due to the negligible evaporation rate at room temperature (i.e. away from any phase transition). We identify local negative and positive temperature variations distributed in 50–80 µm width strata, alternating cold and warm zones synchronously with the applied deformation. The temperature variations observed upon shear strain oscillations in the viscous regime are significant (about ΔT ± 0.2 °C) reproducible and reversible; this thermo-mechanical coupling works without dissipation revealing the ability of the liquid to convert the shear energy in non-uniform thermodynamic states.

## Results

**Figure 1:**
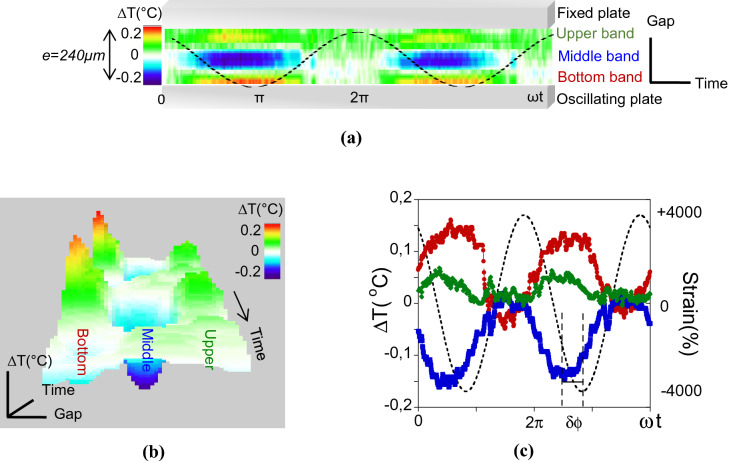
By applying a low frequency shear mechanical stimulus (~ Hz), the liquid emits a modulated thermal signal synchronous with the stimulus. (**a**) Real-time mapping of the temperature variation of the PPG-4000 confined in a 240 µm gap (gap view) excited with a low frequency oscillatory shear strain (room temperature measurements at ω = 0.5 rad/s and γ = 4,000% gathering about ~ 800 frames, alumina substrate). Black line is an eye guide for the applied strain. (**b**) 3D view of the thermal behaviour of Fig. 1a. (**c**) Temperature variations of the bottom, middle and upper bands displayed in red ( ), blue ( ) and green ( ) respectively. The dotted line is an eye guide for the applied shear strain. δφ is the phase shift between the thermal and strain wave. The zero ΔΤ is defined relative to the observation window.

**Figure 2:**
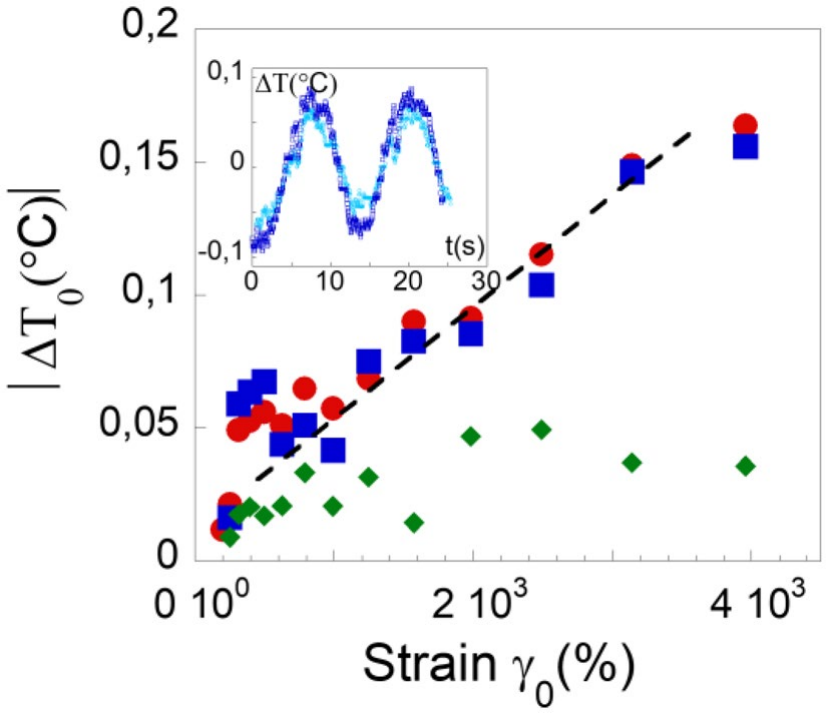
Strain dependence of the maximum of the temperature variation in absolute units ∣*ΔT*_*0*_(°C)∣. Sample: PPG-4000 at gap thickness 0.240 mm, ω = 0.5 rad/s, for bottom “hot” band: ( ), middle “cold” band: ( ) and upper band: ( ) respectively—measurements below 150% are below the accuracy. The insert illustrates the thermal waves for shear strain values 2,500% (light blue points) and 4,000% (blue points) of the middle band (0.240 mm gap, ω = 0.5 rad/s).

Figure 1a,b display the real-time thermal mapping of the low molecular weight polypropylene glycol (PPG-4000) recorded during two oscillatory periods (*ω* = 0.5 rad/s) at a shear strain amplitude *γ* = 4,000%. This representation overviews the instant state of the system at different stages of the mechanical deformation. The thermal image shows the periodic emergence of coexisting hot and cold zones distributed in approximately three thermal bands (Fig. 1a). The bands exhibit opposite thermal behaviours. While the middle band is cooling down, the neighboured ones are heating up. This leads to a temperature compensation in the liquid volume (Fig. 1a,c). These thermal changes are in advance with the applied strain wave (Fig. 1c), the phase shift being approximately *π/4* (*δφ*_*middle*_ ~ 51° ± 2°, *δφ*_*bottom*_ ~ 46° ± 2.5° and *δφ*_*upper*_ ~ 48.25° ± 2°, for the middle, bottom and upper bands respectively). The applied slow dynamics exclude a coupling with time-dependent processes such as the viscoelastic relaxation time (for glycerol *τ*_*relax*_ ~ 10^−9^ s)^13^. The nearly instant thermal signal (Fig. 1) and the linear-like strain-dependence of the amplitude of the thermal waves (Fig. 2) are strong indicators for a direct coupling with the mechanical shear strain which is the only energy source. The compensation between cold and hot waves indicates that the shear waves propagate adiabatically with no time for heat exchange in the medium. The reversibility of the thermal modulation rules out mechanisms related to slow thermal conduction processes. Finally, reversible temperature changes induced mechanically are known in solids as the thermo-(shear)elastic effect^17^.

Figure 2 shows the strain dependence of the temperature variation (amplitude maximum in absolute units) in the three coexisting thermal bands identified in Fig. 1. Cold and hot waves vary in opposite way with increasing strain amplitude (becoming “cooler” and “warmer” respectively). This representation highlights the superposition of “hot” and “cold” values in a nearly exact compensation confirming that the system works adiabatically (without energy external transfer). The inset of Fig. 2 illustrates the thermal waves at two different strain values (for the same thermal band). The temperature variation is not measurable at low strain and increases linearly with the strain amplitude above γ > 200% reaching a maximum variation of ~ 0.2 °C at 4,000% (10 times over the accuracy). The linear relation holds true for the two thermal bands of opposite behaviour closer to the moving surface, while the evolution for the band farthest from the moving surface is nearly independent of the strain amplitude.

**Figure 3:**
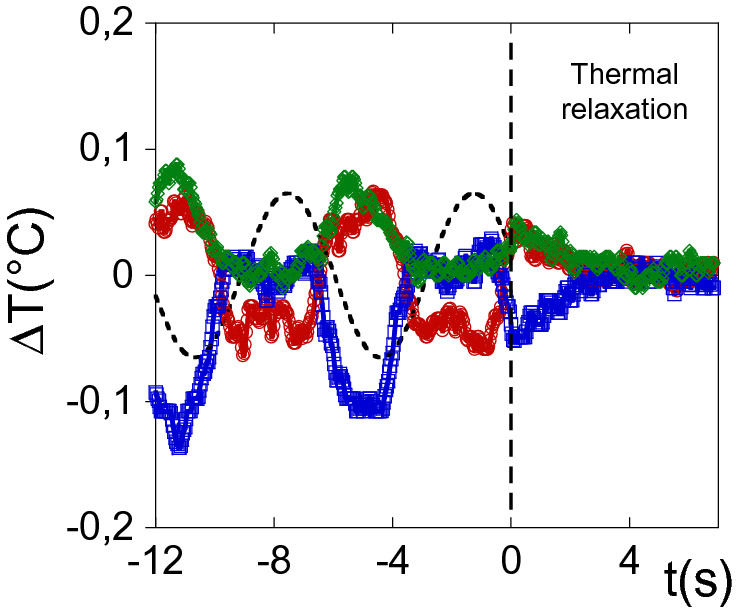
Example of thermal relaxation after an oscillatory shear strain. Red, green and blue data points represent bottom, middle and upper bands of the gap respectively. The dotted line is a guideline for the strain wave. The time zero is the origin of times of the relaxation process at the onset of the shear strain stop. Measurements carried out on PPG-4000 at 0.240 mm gap thickness γ_0_ = 4,000%, ω = 1 rad/s and stopped at γ = 1,200%, alumina substrate.

Once the applied strain is switched off, a thermal relaxation process is observed (Fig. 3). A decrease of the temperature is observed for the hot bands while the temperature of the cold bands simultaneously increases. The main relaxation times (*t*_*relax*_) are about several seconds (typically about 2–10 s modelled by *ΔΤ*(*t*) = *ΔT*_*0.*_*exp*(*−t/t*_*relax*_)) depending on when the stop is triggered with respect to the strain. The thermal relaxation occurs simultaneously for every band, regardless the prior temperature variation. This process is at variance with a dissipative heat transfer process from a hot to a cold source. Cold and hot bands relax from non-equilibrium configurations indicating a prior increase of the liquid internal energy under shear strain. The thermal relaxation reveals the release of the excess internal energy.

**Figure 4:**
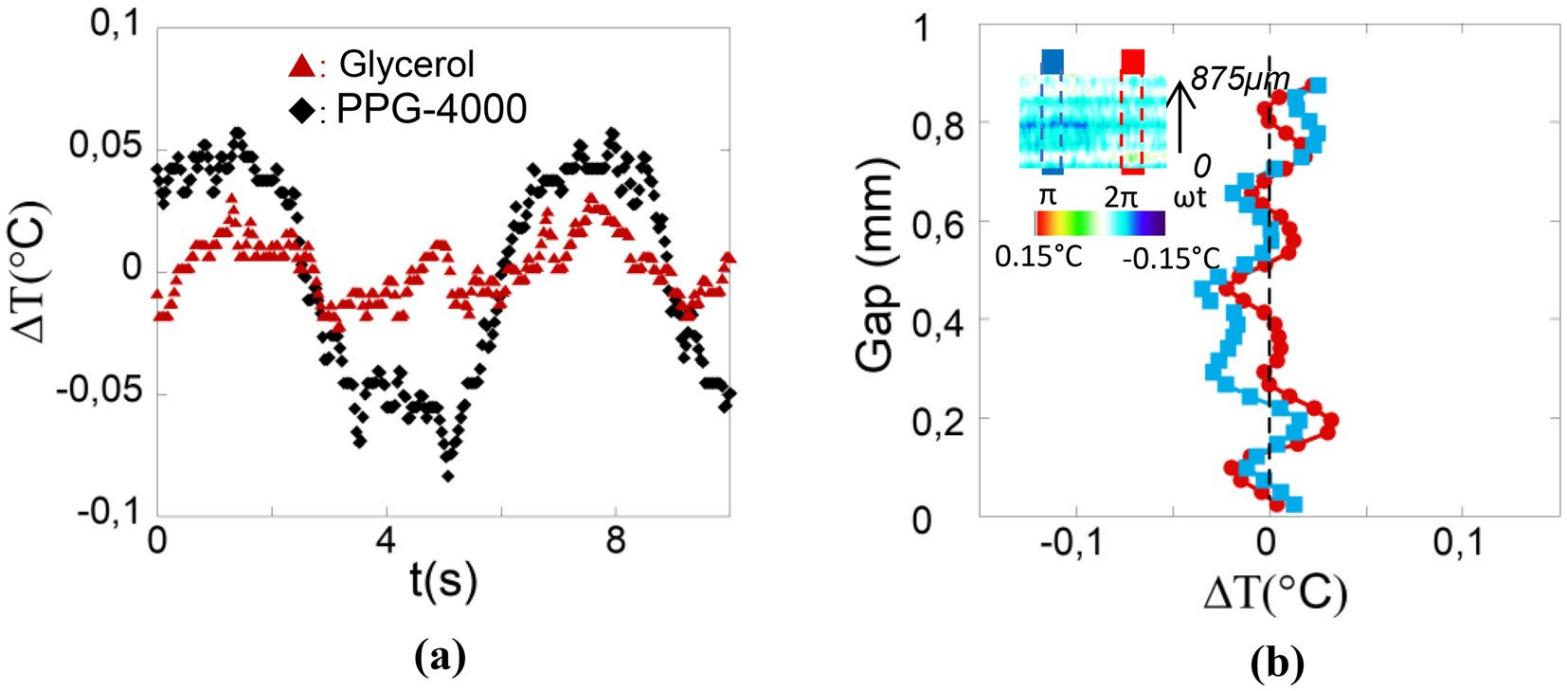
(**a**) Comparison of the thermal behaviour upon shear strain oscillation of glycerol [dark red triangles ( )] and PPG-4000 [black lozenges (◆)]. Identical experimental conditions: (bottom) thermal band at 0.240 mm gap thickness, ω = 1 rad/s and γ_0_ = 2,500%. (**b**) Gap thermal profile (Oz) showing the generation of multiple thick thermal waves at large gap thickness (0.875 mm), glycerol at ω = 1 rad/s and γ = 650%. Red circles ( ) and light blue squares ( ) display the temperature along the gap corresponding to the liquid areas surrounded by red and blue dotted lines in the inset (~ 400 frames).

The glycerol exhibits a similar thermal behaviour upon applying shear strain stress (Fig. 4a), but with a weaker variation compared to PPG-4000 (ΔΤ_max_ ~ 0.12 °C). This weaker response can be interpreted by the key role of the total entropy (intermolecular, conformational). Glycerol is a molecular liquid with symmetrical geometry whereas the low molecular weight PPG-4000 is an oligomer, meaning increased conformational entropy and multiple intermolecular interactions per molecule. Thermal bands are identified with varying gaps from 45 μm. More precisely three thermal bands seems to be the rule for various gap thickness between 150 and 500 μm. It is systematically observed that the bands oscillate in opposite temperature variation implying that the temperature compensation is essential for the mechanism. The spatial position of each band is not fixed along the gap thickness. Above 500 µm, multiple bands (> 3) appear hardly observable being “diluted” in the width of the gap (Fig. 4b and insert). The thermal mapping (Figs. 1a, 4b), the temperature graph (Fig. 1c) and the strain dependence graph (Fig. 2) indicate that the upper band (farthest band from the moving surface) exhibits the weaker thermal signal with respect to the other ones. The thermo-mechanical effect primarily takes place near the motion surface (strain source) which means that the energy might not necessarily propagate along the whole gap.

## Discussion

How to understand that viscous liquids convert a low frequency shear strain in synchronous thermal waves? Such a collective property is unexpected considering viscoelastic relaxation times (1/ω ≪ τ_relax_). Furukawa and Tanaka^18^ suggest that liquids can be compressible by shear flow in order to explain shear banding or cavitation. The theory “does not involve viscoelastic or structural relaxation” but supposes a coupling between flow and relaxation times of slow density fluctuations (of highly viscous liquids like metals, earth’ mantle, viscous polymers)^18^. A comparison with the present experimental conditions rules out since the foreseen shear rates, about 10^6^ s^−1^, are much higher than the inverse of the timescales probed in the low frequency range.

A mechanism alternating compressed-stretched states in volume and time seems plausible; the “stretched” state generates the cold area while the “compressed” state generates the hot. These two thermodynamic states compensate dynamically: i.e. the system does not exchange with the environment (*dQ* = 0). The liquid converts the shear wave energy in localized cold and hot temperature bands without external energy transfer, thus following an adiabatic scheme that can repeat endlessly. We also showed that the thermal signal increases linearly with the strain amplitude for slow dynamics (ω = 0.5 rad/s) highlighting a thermoelastic behaviour that is usually expected in solids only. In solids upon applying a periodic strain (load), periodic thermal changes exhibit temperature oscillations in “opposite” phase with the applied strain^17^. The temperature variation is linear with the applied stress *Δσ* in agreement with the strain dependence observed for the liquid PPG at moderate strain values^17^. However a direct comparison of the temperature variation *ΔΤ* in solid thermoelastic processes cannot be made since the shear stress *Δσ* cannot be measured in the hydrodynamic regime of the liquid. Therefore then comparison is done on basis of the strain equivalence. The linear relationship (Fig. 2) is a strong indication of a thermal mechanism occurring as soon as the smallest shear strain values; i.e. inherent to an elastic property of the liquid. The proportionality between the relative temperature variation *ΔΤ/T* and the mechanical deformation (at low shear strain) defines a dimensionless constant *Θ*_*shear*_(*ΔT*,* γ*) = (*ΔΤ/Τ*)/*γ*. This constant quantifies the relative variation of temperature by (shear) stretching unit. These values are about *Θ*_*strain*_ = (1.20 ± 0.12)*10^–5^ for the hot band (red points) and about *Θ*_*strain*_ = − (1.05 ± 0.12)*10^–5^ for the cold band (blue points) at room temperature. There is no equivalence in liquids since they are not supposed to support shear waves. A thermo-(strain)elastic equivalent can be evaluated for metals: *Θ*_*elongational*_ = (*ΔΤ/T*)/*E* ≅ 0.45 where *E* is the elongational strain in the case of steelsheets submitted to cycle strain fatigue^19^. The constant *Θ* is about four decades higher for the metal indicating that a much higher and always positive temperature variation originating from a different and purely dissipative mechanism (moving defects) in the case of solids.

The nearly instantaneous conversion of the shear deformation energy in coexisting thermal states, implies that density (thermal) fluctuations and liquid interactions are long-range correlated. The shear stress acts down to intermolecular forces inducing instant complementary positive and negative stress variations (without external heat transfer). These non-uniform temperatures are accessible at high strain rates (*γ* > 200%); i.e. in highly non-equilibrium conditions for which the Second Law of thermodynamics does not apply. Under local potential (stress, surface vicinity, etc.), correlated systems typically do not relax to the equilibrium where the injected mechanical energy can be adiabatically transformed generating heating and cooling^20^. Mesoscale liquids must be rather treated as long range shear-elastically correlated systems that reach nearly stable non-equilibrium states. In this frame the liquid elasticity does emerge probably in a similar way as for non-affine models for amorphous systems or disordered solids^21,22^. The present thermal effects highlight exacerbated local excess (compressed state) and deficiency (stretched state) in energy of a strongly non-equilibrium state.

Going back to the definition of shear modes in liquids following Frenkel^9^, liquids would support both longitudinal and transverse waves only above *ω* > *ω*_*F*_, where *ω*_*F*_ describes short interactions (a particle jump) in the GHz range that are mechanically inaccessible and irrelevant for the present study. Trachenko et al.^15,16,23^ revisited the Frenkel’s kinetic theory of liquids. They reconsidered the concept of short-range interaction and introduced the notion of propagating solid-like shear waves defining a k-gap dispersion relation  $\omega =\sqrt{{c}^{2}{k}^{2}-\frac{1}{4{\tau }^{2}}}$ where *c* is the sound velocity and *τ* the time during which the stress releases^15,16,23^. This dispersion law indicates that the propagation of solid-like shear waves is made possible for k > k_g_ = 1/(2c·τ) (real solutions). The present observations carried out at mesoscopic scale provide experimental evidence in this direction. As mentioned in^16^, “*we consider a liquid as collection of dynamical regions of characteristic size c.τ where the solid-like ability to support shear waves operate*”. These dynamical regions might coincide with the thermal bands. The propagation length of the shear waves *d*_*el*_ = *c*·*τ* corresponds in the case of glycerol to *d*_*el*_ ~ 3 μm (*c* = 3000 m/s and *τ* = 10^−9^ s given for density fluctuations at equilibrium^13^). The predicted propagation length is smaller than the width of the elementary hot and cold bands (~ 25–80 μm) which might indicate more extended “correlated” interactions. The prediction of a finite propagation of the solid-like shear wave might explain why the liquid splits in several thermal bands, *d*_*el*_ being the ultimate liquid length supporting the shear wave. Over this limit, an interface is created, enabling the conditions for a new shear mechanism to take place and producing new elementary (thermal) shear bands, each delimited by the finite propagation length. This scheme is also in agreement with the vanishing of the induced thermal effect away from the surface in motion (shear source). Figure 2 shows that the temperature of the furthest band from the shear plate is nearly unaffected by the strain amplitude. Measurements at large volumes (inset Fig. 4b) show a “dilution” of the thermal oscillations, towards a possible thermal homogenization. These observations reinforce the idea that the liquid properties are not homogeneous, but dependent on the scale at which they are probed^1^, in agreement with a finite shear wave propagation^14–16,23^ and the identification of a scale-dependent low frequency shear-elasticity^2–8^. The correlated liquid molecules nearly instantaneously adapt the thermodynamic state according to the applied strain. This thermal effect identified far away from any critical point (here the glass transition), suggests a generic thermo-mechanical property. These are new insights for the microfluidic understanding. Important applications for thermal control in micro-devices and heat sinks can be foreseen using and controlling these newly uncovered thermo-mechanical liquid properties.

## Methods

The thermal emission was recorded using an infrared sensor equipped with a macro-lens focussing the liquid gap in between the two plates. The frame rate was 27 Hz—and the depth of field (DOF) is ~ 0.85 mm. The thermal accuracy is ± 0.020 °C. For temperatures near ambient temperature, the emitted radiation is in the near infrared range (700–1000 μm). Based on the Stefan-Boltzmann law:

E $={\varepsilon }_{m}\sigma ({T}^{4}-{T}_{c}^{4})$ where *E* is the energy flux, *ε*_*m*_ the emissivity, *σ* the Stefan–Boltzmann constant, *T* the temperature and *T*_*c*_ the environment temperature. The thermal pictures have been corrected from the static thermal environment by subtracting the median value measured at rest prior the dynamic measurements. An example of thermal mapping of liquid at rest is displayed on Scheme 1. The real-time mapping were obtained by construction of a kinetic image made of the succession of each frame (1 frame/0.037 s).

The liquid was confined between two disk-like α-Alumina surfaces to increase the interaction between the surface and the liquid molecules^7,8^. The surfaces have been heat treated beforehand to remove any organic component and moisture. This procedure and the high energy surfaces ensure the total wetting. Alumina has a relatively low thermal conductivity (28–35 W/m^−1^ K^−1^ at room temperature) compared to metallic substrates (Aluminum ~ 230 W/m^−1^ K^−1^). The liquid is itself poorly thermally conductive (0.30 W/m^−^1 K^−1^). The geometry of our setup is a conventional plate-plate one. Shear strain (γ = δl/e, where δl is the displacement and e the gap thickness) is applied by oscillating the bottom surface (Fig. 1). Shear strain is a dimensionless unit and in this paper is presented as a percentage. The upper surface is coupled with a sensor, which measures the shear stress (torque) transmitted by the liquid. Keithley multimeters record strain and stress mechanical waves with high accuracy. Both mechanical and thermal measurements were recorded simultaneously.

Glycerol and polypropylene glycol (PPG-4000, M_n_ = 4,000) are nearly black bodies in mid- and near-infrared radiations. Mechanical and thermal behaviours were systematically probed for gap thickness from 500 μm down to 85 μm and at a frequency range from 0.5 to 5 rad/s, at room temperature away from any critical point (glass transition temperatures for glycerol and PPG are − 93 °C and − 73 °C respectively).

## Data Availability

The datasets generated and analysed during the current study are available from the corresponding author on reasonable request.
